# Targeting FBXO22 enhances radiosensitivity in non-small cell lung cancer by inhibiting the FOXM1/Rad51 axis

**DOI:** 10.1038/s41419-024-06484-1

**Published:** 2024-01-31

**Authors:** Yunshang Chen, Yun Zhou, Xue Feng, Zilong Wu, Yongqiang Yang, Xinrui Rao, Rui Zhou, Rui Meng, Xiaorong Dong, Shuangbing Xu, Sheng Zhang, Gang Wu, Xiaohua Jie

**Affiliations:** 1 Cancer Center, Union Hospital, Tongji Medical College, Huazhong University of Science and Technology, Wuhan, 430022 China; 2 Institute of Radiation Oncology, Union Hospital, Tongji Medical College, Huazhong University of Science and Technology, Wuhan, 430022 China; 3 Hubei Key Laboratory of Precision Radiation Oncology, Wuhan, 430022 China; 4 Department of Pediatric Surgery, Union Hospital, Tongji Medical College, Huazhong University of Science and Technology, Wuhan, 430022 China; 5 Department of Obstetrics and Gynecology, Union Hospital, Tongji Medical College, Huazhong University of Science and Technology, Wuhan, 430022 China

**Keywords:** Radiotherapy, Non-small-cell lung cancer

## Abstract

Radioresistance is a major constraint on the efficacy of lung cancer radiotherapy, but its mechanism has not been fully elucidated. Here, we found that FBXO22 was aberrantly highly expressed in lung cancer and that FBXO22 knockdown increased the radiosensitivity of lung cancer cells. Mechanistically, FBXO22 promoted Rad51 gene transcription by increasing the level of FOXM1 at the Rad51 promoter, thereby inducing the formation of lung cancer radioresistance. Furthermore, we found that deguelin, a potential inhibitor of FBXO22, enhanced radiosensitivity in an FBXO22/Rad51-dependent manner and was safely tolerated in vivo. Collectively, our results illustrate that FBXO22 induces lung cancer radioresistance by activating the FOXM1/Rad51 axis and provide preclinical evidence for the clinical translation of this critical target.

## Introduction

Lung cancer is a malignant heterogeneous tumor with the highest mortality rate among cancers worldwide [1]. Non-small cell lung cancer (NSCLC) is the most common pathologic tissue type, with lung adenocarcinoma being the predominant subtype. As an efficient and cost-effective treatment for NSCLC, radiotherapy is estimated to consume 14% of social resources but contribute to 40% of tumor cure rates. As reported, radiotherapy plays a decisive role in the treatment of early inoperable and locally progressive NSCLC, increasing the 5-year local control rate by 8.3% [2, 3] Therefore, identifying molecular markers of radioresistance and developing inhibitors against them is of great clinical value.

Radiotherapy exerts antitumor effects by inducing DNA damage. To protect the integrity of genetic information, tumor cells have evolved a set of complex and tightly regulated mechanisms to recognize and repair damaged DNA [4]. Homologous recombination (HR) is a high-fidelity DNA double-strand break repair mechanism whose abnormal activation is the main contributor to radioresistance [5]. Several HR molecules have been reported to be transcriptionally regulated by transcription factor Forkhead box M1 (FOXM1), including Rad51, the central member of HR. Previous studies have shown that targeting the FOXM1/Rad51 axis reverses temozolomide resistance in recurrent glioblastoma cells and enhances sensitivity to PARP inhibitor in ovarian cancer [6, 7]. In addition, the FOXM1/Rad51 axis protect lung fibroblasts from radiation-induced cell death [8], indicating that this axis may be involved in lung cancer radiosensitivity.

F-box-only protein 22 (FBXO22) is an important component of the SKP1-Cullin-F-box (SCF) E3 ubiquitin ligase. FBXO22 participates in multiple biological processes, such as cellular senescence, cell cycle regulation, and autophagy, by recognizing and targeting different substrate molecules [9]. Several studies have shown that FBXO22 is associated with tumor development and therapeutic response. For instance, FBXO22 promotes melanoma progression and metastasis by upregulating HIF-1α and VEGF [10]. FBXO22-mediated ubiquitination and degradation of BACH1 are implicated in leukemogenesis and stem cell maintenance [11]. In addition, FBXO22 acts as a molecular switch to determine the sensitivity of ER^+^ breast cancers to selective estrogen receptor modulators (SERMs), such as tamoxifen [12]. Notably, knockdown of FBXO22 increases apoptosis induced by DNA-damaging agents such as adriamycin, suggesting that targeting FBXO22 may synergize with radiotherapy [13]. Thus far, the function and molecular mechanisms of FBXO22 in lung cancer radiosensitivity have not been fully elucidated.

In the present study, we found that FBXO22 was aberrantly highly expressed in lung cancer and that high expression of FBXO22 was associated with poor clinical prognosis. FBXO22 silencing increased the radiosensitivity of lung cancer cells. Mechanistically, FBXO22 facilitated transcriptional activation of the homologous recombinase Rad51 through upregulation of the transcription factor FOXM1, thus promoting DNA damage repair and lung cancer radioresistance. Furthermore, deguelin was screened as a small molecule inhibitor of FBXO22 by using the CMap database and was confirmed to be radiosensitizing in an FBXO22/Rad51-dependent manner. Collectively, our study provides new therapeutic insights to overcome lung cancer radioresistance.

## Materials and Methods

### Cell culture

All cell lines (BEAS-2B, A549, H1299, H460, and H1975) were purchased from the American Type Culture Collection (ATCC) and cultured in DMEM/F12 or RPMI-1640 containing 10% fetal bovine serum at 37 °C in 5% CO_2_. No signs of mycoplasma contamination were found in any of the cell lines and all cells were identified by using short tandem repeat profiling (STR).

### RNA interference

Small interfering RNAs (siRNAs) specifically targeting FBXO22 and FOXM1 were purchased from JTSBIO Co., Ltd., and transfected using Lipofectamine RNAiMAX reagent (Invitrogen, USA) at a cell density of 30-40%. The sequences of the siRNAs were as follows:

Scramble siRNA: 5′-UUCUCCGAACGUGUCACGU-3′;

SiFBXO22#1: 5′-GUUCGCAUCUUACCACAUA-3′;

SiFBXO22#2: 5′-GCACCUUCGUGUUGAGUAA-3′;

SiFOXM1#1: 5′-GGAAGCGCAUGACUUUGAA-3′;

SiFOXM1#2: 5′-GCAAGAUCCUGCUGGACAU-3′.

### Plasmid transfection

The overexpression plasmids SFB-FBXO22, Flag-Rad51, and Flag-FOXM1 were purchased from Paivi Biosciences Inc., and cells were transfected using Lipofectamine 2000 reagent (Invitrogen, USA) at a cell density of 70–90%. The medium was replaced with complete medium after 4 h. Cells were collected 24 h after transfection for subsequent experiments.

### Antibodies and reagents

The primary antibodies and reagents used in this study included rabbit anti-FBXO22 (Proteintech, 13606-1-AP, 1:2000), mouse anti-GAPDH (Proteintech, 60004-1-Ig, 1:10,000), rabbit anti-Rad51 (Abcam, ab133534, 1:2000), rabbit anti-TOPBP1 (Proteintech, 23340-1-AP, 1:500), rabbit anti-XRCC3 (Proteintech, 18494-1-AP, 1:500), rabbit anti-BARD1 (Proteintech, 22964-1-AP, 1:500), rabbit anti-FOXM1 (Proteintech, 13147-1-AP, 1:1000), mouse anti-Flag (Abclonal, AE005, 1:2000), rabbit anti-LIN9 (Proteintech, 17882-1-AP, 1:500), Harmine (MCE, HY-N0737A, China) and deguelin (MCE, HY-13425, China).

### CRISPR-Cas9

gFBXO22 and lenticrspr V2_puro vectors were purchased from Paivi Biosciences Inc. A549 cells were infected with viruses carrying cas9 and gRNA for 48 h and cultured in DMEM/F12 containing 2 μg/mL puromycin for one week. Cells were seeded individually into 96-well plates to form monoclones. The efficacy of FBXO22 knockdown was verified by Western blotting. The sequence of gFBXO22 was GCACACACUCCCUCCAUAAG.

### Western blot analysis

Cells were collected and lysed with NETN lysis solution containing 20 mM Tris-HCl (pH 8.0), 100 mM NaCl, 1 mM EDTA and 0.5% Nonidet P-40. The protein extracts were separated by SDS-PAGE and transferred to PVDF membranes. After being blocked with 5% skim milk, incubated overnight at 4 °C with primary antibody, and incubated with secondary antibody the next day, the membranes were imaged by using a UVP ChemiDocIt 510 chemiluminescence instrument. Uncropped Western blots were presented in the Supplemental File.

### Real-time quantitative polymerase chain reaction (qRT-PCR)

Total cellular RNA was extracted according to the instructions of the RNA Extraction Kit (Omega, USA) and reverse-transcribed into cDNA using HiScript III-RT SuperMix (Vazyme, China). qRT-PCR was performed with ChamQ SYBR qPCR Master Mix (Vazyme, China). The primer sequences are shown in Table S1.

### Immunohistochemical (IHC) staining

Lung adenocarcinoma tissue microarrays were purchased from Shanghai Outdo Biotech (Product number: HLugA180Su07). IHC experiments and score calculations were performed as described previously [14]. Each image was individually scored by three pathologists with blinding. The IHC primary antibodies used in this study included rabbit anti-FBXO22 (Proteintech, 13606-1-AP, 1:400), rabbit anti-Rad51 (Abcam, ab133534, 1:1600), and rabbit anti-Ki67 (Abcam, ab16667, 1:1200).

### RNA sequencing (RNA-seq) and the connectivity map (CMap)

Scramble and SiFBXO22 were transfected into lung cancer A549 cells, and 48 h later, the cells were collected with TRIzol lysis buffer. The samples were sent to BGI-Shenzhen, China, for RNA sequencing. The specific procedure of RNA sequencing was as previously described [14]. The raw sequencing data in this study have been uploaded to the GEO database (https://www.ncbi.nlm.nih.gov/geo/) with registration number GSE235374. The 300 differentially expressed genes with the most significant changes in RNA-seq (including 150 upregulated genes and 150 downregulated genes) were matched with the reference dataset in the CMap database to obtain the candidate inhibitors of FBXO22.

### Luciferase assay

The Rad51 promoter sequences were cloned into the pGL3 vector to construct the reporter plasmids, and TAATCA located at −764 to −758 was mutated to TGCTCA based on the previous report [6]. Cells were seeded in 12-well plates. After transfection of the corresponding siRNAs and luciferase reporter plasmids, the activation of the reporter genes was detected by using the Dual-Luciferase Reporter Gene Assay Kit (Beyotime, RG027). The renilla luciferase was used as an internal reference to calculate the relative luciferase activity.

### ChIP-PCR analysis

ChIP Assay Kit (Beyotime, P2078) was utilized to perform ChIP-PCR experiments. Briefly, the DNA and proteins in cell samples were cross-linked by 1% formaldehyde solution and chromatin was fragmented to 200-800 bp by ultrasound. The chromatin fragments were then enriched using FOXM1 antibody (Proteintech, 13147-1-AP) or IgG (Proteintech, 30000-0-AP) and incubated overnight at 4 °C. After washing, decrosslinking, and DNA purification, the samples were measured by PCR with three different primers designed for Rad51 promoter. The primer sequences are shown in Table S2.

### Colony formation assay

After trypsin digestion and cell counting, the cells were seeded in six-well plates at 200, 400, 1000, 3000, and 6000/well, physically irradiated at 0, 2, 4, 6, and 8 Gy after attachment, and then cultured in a 37 °C incubator for 14 days. After methanol fixation and crystal violet staining, the colonies with more than 50 cells were counted.

### Neutral comet assay

Neutral comet assays were performed by using a Comet Kit (Trevigen, USA) according to the manufacturer’s specifications. In brief, cells were mixed with LMAgarose and smeared onto Comet slides 4 h after irradiation with 6 Gy. The slides were then immersed in lysis buffer and neutral electrophoresis solution and subjected to horizontal electrophoresis for 1 h. After DNA precipitation and SYBR Gold staining, photographs were taken under a fluorescence microscope, and the results were analyzed with Cometscore 2.0 software.

### Immunofluorescence staining

Cells were exposed to 6 Gy irradiation before the experiment started. After being fixed with 4% paraformaldehyde, ruptured with 0.2% Triton X-100 and blocked with 5% BSA, the cells were incubated with rabbit anti-γH2AX (Abcam, ab81299, 1:800) and rabbit anti-Rad51 (Abcam, ab133534, 1:800) overnight at 4 °C. Fluorescent secondary antibody incubation and DAPI staining were performed the next day. Photographs were taken with a laser confocal microscope, and the foci-positive cells with more than 10 foci in the nucleus were counted.

### Tumor xenograft experiment

Four-week-old female BALB/c nude mice were provided by Changzhou Cavens Laboratory Animal Co., Ltd. The mice were randomized into groups of 6 based on previous experience. 5 ×10^6^ A549 cells were inoculated into the axilla of the right upper limb of each mouse. The mice in the radiation groups were subjected to 10 Gy local irradiation when the tumors reached 100 cm^3^. For deguelin treatment, the mice were orally administered deguelin (4 mg/kg) or solvent every other day. The tumor length and width were measured every 3 days with blinding, and the tumor size was calculated according to the formula volume = length x width^2^/2.

### Statistical analysis

In this study, The Student’s t test (two-tailed) and ANOVA were used respectively to examine the statistical differences between two groups and multiple groups. The differential expression of FBXO22 in lung cancer and adjacent tissues were analyzed by the Chi-square test, and the Kaplan-Meier method was utilized to evaluate the prognostic relevance of FBXO22. The data are presented as the means ± SDs unless otherwise stated. All data meet the assumptions of the tests and statistical tests are justified as appropriate. *P* < 0.05 was considered to indicate statistical significance (* *P* < 0.05, ** *P* < 0.01, *** *P* < 0.001).

## Results

### FBXO22 is overexpressed and predicts poor clinical outcomes in lung cancer

**Fig. 1:**
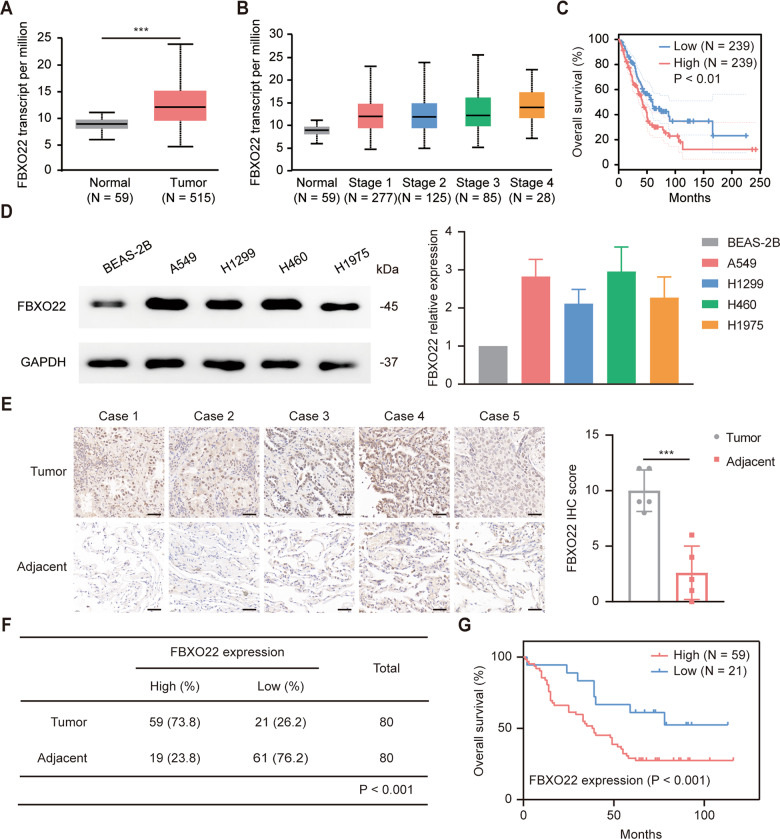
Overexpression of FBXO22 predicts poor prognosis in lung cancer. **A** Data from the UALCAN database indicating that FBXO22 is more highly expressed in lung adenocarcinoma tissue than in normal lung tissue. *** *P* < 0.001. **B** The expression of FBXO22 tends to increase with increasing pathological grade according to the UALCAN database. **C** Relationship between FBXO22 expression and overall survival of lung cancer patients in the GEPIA database. ** *P* < 0.01. **D** Left panel: protein levels of FBXO22 in different cell lines were detected by Western blotting. Right panel: The relative protein level in each cell line in comparison to BEAS-2B cells was calculated by ImageJ software (*n* = 3). **E** Representative images of IHC staining of tumor and adjacent tissues from five lung adenocarcinoma patients and statistical histograms of the IHC score. *** *P* < 0.001 (n = 5). Scale bar: 20 µm. **F** IHC staining was performed on a lung adenocarcinoma microarray. The expression level of FBXO22 is presented in the statistical table. *** *P* < 0.001. **G** The correlation between FBXO22 expression and the overall survival of patients in the microarray was assessed by the Kaplan‒Meier method. *** *P* < 0.001.

To explore the role of FBXO22 in lung cancer, we searched the UALCAN database (https://ualcan.path.uab.edu/) and found that the expression of FBXO22 was significantly higher in lung cancer tissues than in normal lung tissues and that it tended to increase with increasing pathological grade, suggesting that overexpression of FBXO22 was associated with worse pathological stage and poor prognosis (Fig. 1A, B). Findings from the GEPIA database (http://gepia.cancer-pku.cn/) also indicated that overall survival was shorter in lung cancer patients with higher FBXO22 expression (Fig. 1C). To validate the results from the database, we first examined the protein levels of FBXO22 in different cell lines. As expected, the expression of FBXO22 was generally upregulated in lung cancer cell lines (A549, H1299, H460, and H1975) compared with normal human bronchial epithelial BEAS-2B cells (Fig. 1D). Additionally, immunohistochemical (IHC) staining of paired tissue samples collected from our lung adenocarcinoma patients also indicated that the expression of FBXO22 was dramatically elevated in lung cancer tissues (Fig. 1E). To further clarify the clinical relevance of FBXO22, we conducted IHC staining of a lung adenocarcinoma microarray and found that 73.8% of the lung cancer tissues showed high expression of FBXO22, while 76.2% of the adjacent tissues showed low expression of FBXO22, and the difference was statistically significant (Fig. 1F). Kaplan-Meier survival analysis revealed that the median survival of patients with high FBXO22 expression was 38.5 months, which was much shorter than that of patients with low FBXO22 expression (not reached) (Fig. 1G). In conclusion, FBXO22 is highly expressed in lung cancer, and its expression is a predictor of poor prognosis.

### FBXO22 silencing increases the radiosensitivity of lung cancer cells

**Fig. 2:**
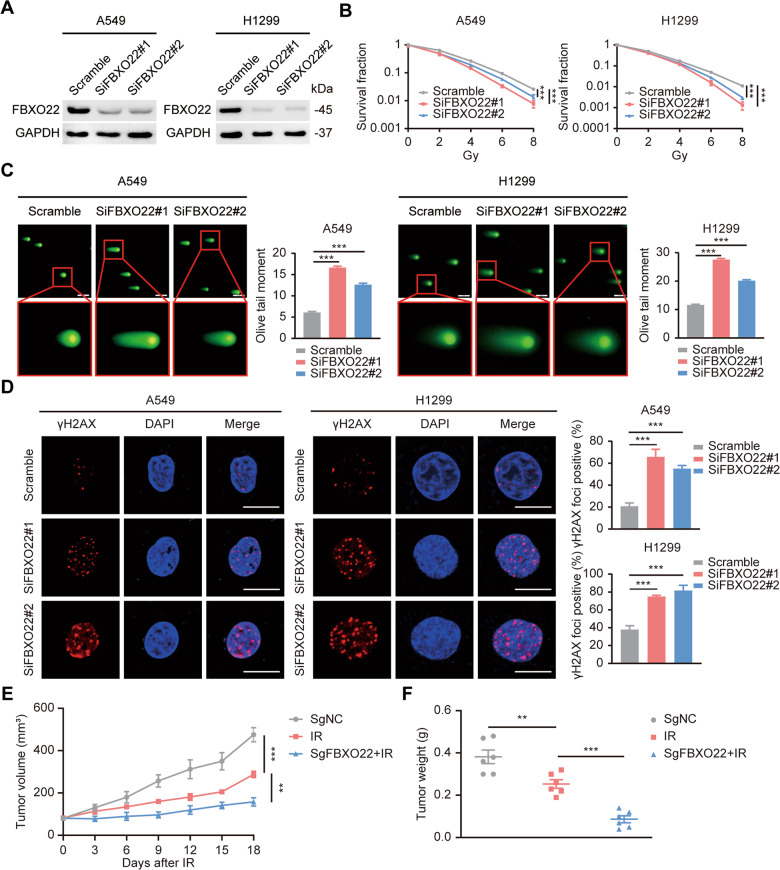
FBXO22 silencing increases the radiosensitivity of lung cancer cells. **A** FBXO22 was effectively silenced by two siRNAs with different sequences. **B** The control and FBXO22-knockdown lung cancer cells were physically irradiated with 0, 2, 4, 6, and 8 Gy, and the cell colonies in each group were counted after 14 days. *** *P* < 0.001 (*n* = 3). **C** Lung cancer cells in the indicated groups were subjected to 6 Gy irradiation followed by a neutral comet assay. Representative images of fluorescence staining and statistical histograms of the olive tail moments are shown. The data are presented as the mean ± SEM. *** *P* < 0.001 (*n* = 100). Scale bar: 20 µm. **D** Control and FBXO22-silenced lung cancer cells were collected for γH2AX immunofluorescence staining after receiving 6 Gy physical irradiation. The γH2AX foci-positive cells in each group were counted under a fluorescence microscope. *** *P* < 0.001 (*n* = 3). Scale bar: 10 µm. **E** The tumor xenograft model was constructed and the growth curves of xenograft in each group were depicted. Data are presented as mean ± SEM. ** *P* < 0.01, *** *P* < 0.001 (*n* = 6). **F** The tumor weights of each group were recorded and are shown as the mean ± SEM. ** *P* < 0.01, *** *P* < 0.001 (*n* = 6).

Previous studies have shown that FBXO22 inhibition increases apoptosis induced by DNA-damaging agents, implying that FBXO22 may be a potential sensitization target for radiotherapy [13]. To clarify the biological role of FBXO22 in lung cancer radiotherapy, we silenced the expression of FBXO22 with two different methods (SiRNA and CRISPR-Cas9 technology) (Fig. 2A and Fig. S1A). Functional experiments showed that cells with FBXO22 knockdown had reduced survival fraction, prolonged comet tail and an increased number of γH2AX foci compared with the control group, suggesting an augmented radiosensitivity of the FBXO22-silenced cells (Fig. 2B–D and Fig. S1B–D). More importantly, in the tumor xenograft model of nude mice, sgFBXO22 combined with IR showed a notable reduction in tumor size compared with the IR group, indicating that FBXO22 knockout also exerts a synergistic effect of radiotherapy in vivo (Fig. 2E, F and Fig. S1E). All of the above experiments confirmed that genetic silencing of FBXO22 dramatically enhanced the radiosensitivity of lung cancer cell in vitro and in vivo.

### The homologous recombinant enzyme Rad51 is the key downstream substrate of FBXO22

**Fig. 3:**
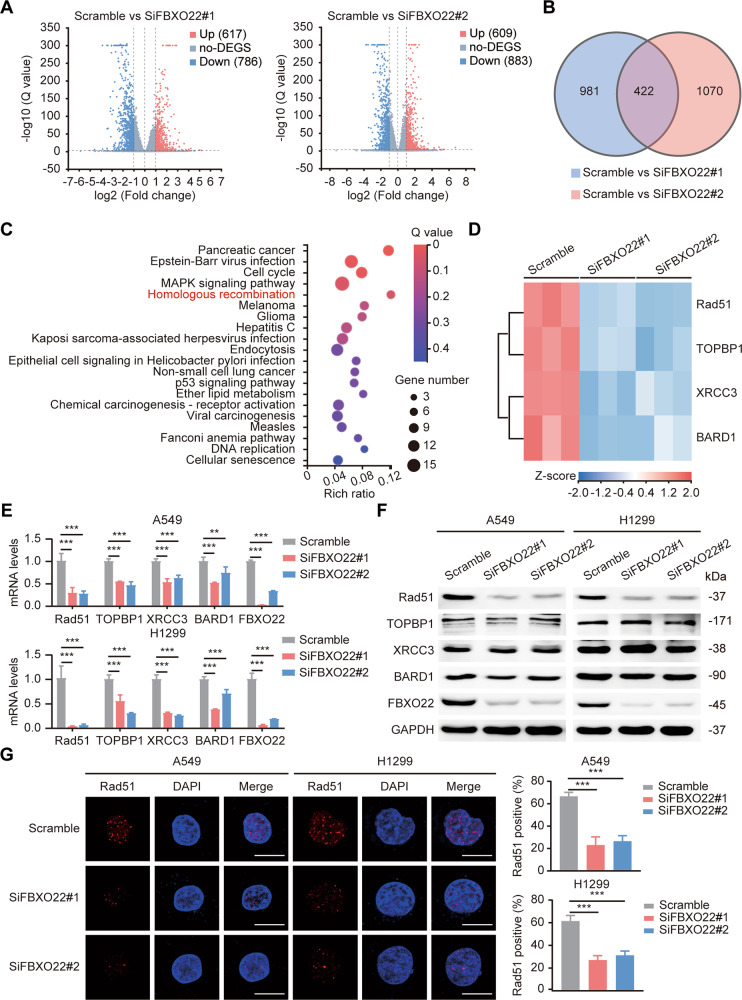
The expression of Rad51 is downregulated in FBXO22-deleted lung cancer cells. **A** The two siRNAs targeting FBXO22 resulted in upregulation of 617 and 609 genes and downregulation of 786 and 883 genes, respectively, with Q value < 0.001, fold change absolute value ≥ 2. Q value is the adjusted *P* value and is used to measure the probability of false positives. Fold change absolute value ≥ 2 represents that the expression level of a certain gene is upregulated to more than two times or downregulated to less than 0.5 times of the control group. **B** A total of 422 intersecting differentially expressed genes were generated by the two siRNAs. **C** KEGG pathway enrichment analysis of the intersecting differentially expressed genes was performed, and the HR pathway was significantly enriched. **D** Clustering heatmap of related genes in the HR pathway. **E** The mRNA levels of the indicated genes in control and FBXO22-silenced A549 and H1299 cells were measured by qRT-PCR. ** *P* < 0.01, *** *P* < 0.001 (*n* = 4). **F** The protein level of Rad51 was significantly reduced in cells with FBXO22 knockdown. **G** Cells were subjected to 6 Gy irradiation and collected for Rad51 immunofluorescence staining. The Rad51 foci-positive cells in each group were counted under a fluorescence microscope. *** *P* < 0.001 (*n* = 3). Scale bar: 10 µm.

To elucidate the downstream mechanism of FBXO22-induced lung cancer radioresistance, we performed RNA-seq in scrambled and FBXO22-silenced A549 cells. As shown in Fig. 3A, the two siRNAs generated 1403 and 1492 differentially expressed genes compared with control cells (Q value < 0.001 and fold change absolute value ≥ 2). KEGG pathway enrichment analysis was conducted on 422 of the intersecting differentially expressed genes, and the enriched pathways were ranked according to the Q value (Fig. 3B, C). We focused on the top five pathways, among which cell cycle, MAPK signaling pathway, and homologous recombination (HR) were reported to be closely associated with radiosensitivity [15, 16]. To further determine the downstream pathway of FBXO22, we analyzed the correlation between the expression of FBXO22 and the relevant molecules in the three pathways mentioned above using the GEPIA database. The results indicated that HR molecules (Rad51, TOPBP1, XRCC3, and BARD1) were all positively associated with FBXO22, in which Rad51 showed the strongest correlation (*R* = 0.5) (Fig. S2A). In contrast, molecules in cell cycle and MAPK signaling pathway were either not correlated (*P* > 0.05) or weakly correlated (*R* < 0.2) with FBXO22 (Fig. S2B, C). Further clustering analysis revealed that HR molecules were all downregulated in FBXO22-deleted cells (Fig. 3D). This conclusion was then confirmed by qRT-PCR (Fig. 3E). We next examined the protein levels of the candidate molecules and found that the expression of Rad51 was markedly decreased with FBXO22 silencing, while other molecules showed no significant changes in expression (Fig. 3F). A subsequent Rad51 foci assay further validated the positive regulatory effect of FBXO22 on Rad51 (Fig. 3G). Thus, FBXO22 is likely to activate HR through the upregulation of Rad51, thereby leading to lung cancer radioresistance.

### FBXO22 upregulates Rad51 in a FOXM1-dependent manner, which in turn induces lung cancer radioresistance

**Fig. 4:**
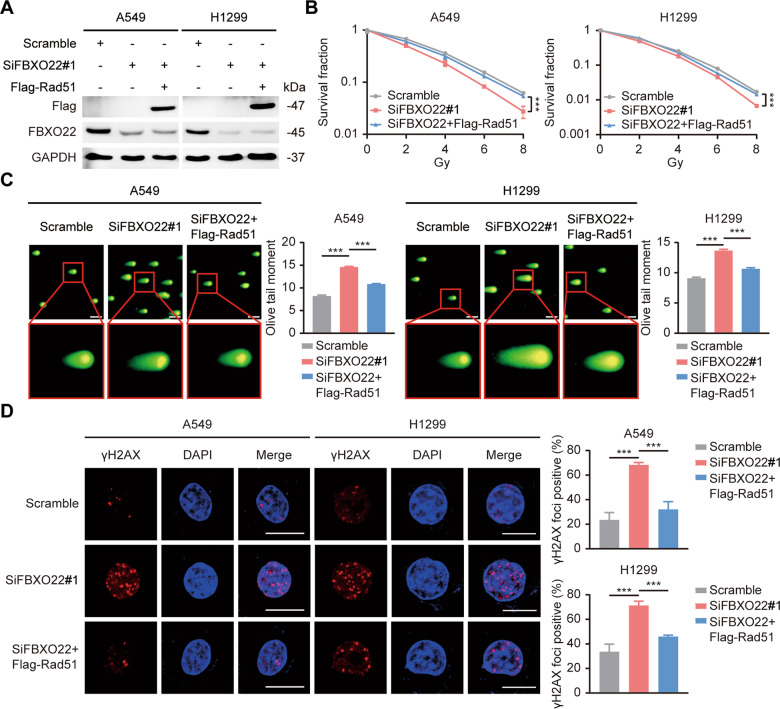
FBXO22 induces lung cancer radioresistance in a Rad51-dependent manner. **A** A549 and H1299 cells were successfully transfected with SiFBXO22 and Flag-Rad51, as verified by Western blotting. **B** Cells in the indicated groups were physically irradiated with 0, 2, 4, 6, and 8 Gy, and the cell colonies were counted and statistically analyzed after 2 weeks. *** *P* < 0.001 (*n* = 3). **C** Representative images of the neutral comet assay and statistical histograms of olive tail moments for each group are presented. The data are shown as the mean ± SEM. *** *P* < 0.001 (*n* = 100). Scale bar: 20 µm. **D** γH2AX foci were stained after 6 Gy irradiation, and the foci-positive cells was counted under a fluorescence microscope. *** *P* < 0.001 (*n* = 3). Scale bar: 10 µm.

To explore the role of Rad51 in FBXO22-mediated radioresistance, a series of rescue experiments were carried out after exogenous overexpression of Rad51 in FBXO22-silenced cells (Fig. 4A). The colony formation assay, neutral comet assay, and γH2AX foci staining all showed that FBXO22 deletion led to increased cellular DNA damage and radiosensitivity and that this effect could be reversed by Rad51 overexpression (Fig. 4B–D). This suggests that Rad51 is essential for the development of FBXO22-induced lung cancer radioresistance.

**Fig. 5:**
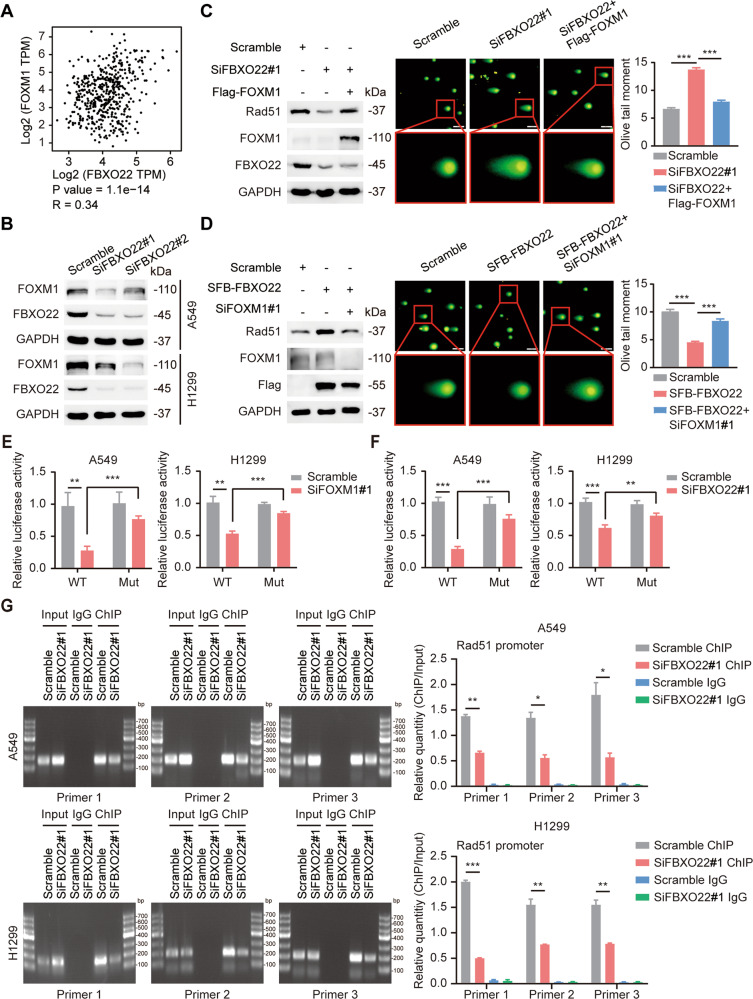
FBXO22 increases the level of FOXM1 at the Rad51 promoter and leads to lung cancer radioresistance. **A** The expression correlation between FBXO22 and FOXM1 in lung adenocarcinoma from the GEPIA database. *** *P* < 0.001. **B** The expression of FOXM1 is downregulated with FBXO22 silencing. **C**, **D** A549 cells transfected with the indicated siRNAs and plasmids were harvested and analyzed by Western blotting and the radiosensitivity of indicated groups was determined by neutral comet assay. Data are presented as mean ± SEM. *** *P* < 0.001 (*n* = 100). Scale bar: 20 µm. **E**, **F** Wild-type and mutant reporter plasmids were transfected in scrambled and FOXM1-silenced (or FBXO22-silenced) lung cancer cells. The relative luciferase activity of each group was calculated using renilla luciferase as an internal reference. ** *P* < 0.01, *** *P* < 0.001 (*n* = 3) **G**. Knockdown of FBXO22 significantly reduced the level of FOXM1 at the Rad51 promoter region. * *P* < 0.05, ** *P* < 0.01, *** *P* < 0.001 (*n* = 3).

How does FBXO22 regulate the expression of Rad51? Since FBXO22 silencing can suppress both the mRNA and protein expression of Rad51, we hypothesized that FBXO22 may affect Rad51 expression by regulating upstream transcription factors of Rad51. To test this conjecture, we first searched the GEPIA database for expression correlations between FBXO22 and the reported Rad51 transcription factors (including FOXM1, TP53, E2F1, E2F4, E2F7, EGR1, CDK12, and CDK13) in lung adenocarcinoma and found that FOXM1 correlated most strongly with FBXO22 (*R* = 0.34, *P* < 0.001) (Fig. 5A and Fig. S3). Consistent with the database results, FOXM1 was downregulated upon FBXO22 silencing and promoted Rad51 expression at the transcriptional and translational levels in lung cancer cells (Fig. 5B and Fig. S4). Importantly, rescue experiments further confirmed that FOXM1 overexpression attenuated the decrease in Rad51 expression and the increase in radiosensitivity caused by FBXO22 deletion; the elevation in Rad51 expression and the reduction in radiosensitivity in response to FBXO22 overexpression were also reversed by FOXM1 silencing (Fig. 5C, D and Fig. S5). In summary, FBXO22 increases the expression of Rad51 in a FOXM1-dependent manner, which in turn triggers lung cancer radioresistance.

As a proliferation-specific oncogenic transcription factor, FOXM1 can directly bind to the promoter of Rad51 to transactivate its expression [6]. Does FBXO22 regulate the expression of Rad51 through this mechanism? To confirm this conjecture, we designed wild-type and mutant luciferase reporter plasmids based on the binding site of FOXM1 to the Rad51 promoter and detected the luciferase activity after co-transfecting them with siRNA targeting FOXM1 in lung cancer cells. The results of the Luciferase assay revealed that FOXM1 silencing markedly inhibited the activity of Rad51 gene promoter, whereas mutation of the binding site significantly reversed this effect, confirming that Rad51 is a direct target of FOXM1 (Fig. 5E). Importantly, FBXO22 silencing similarly attenuated the relative luciferase activity, and this observation was dependent on the direct binding of FOXM1 to Rad51 (Fig. 5F). In addition, ChIP-PCR experiments further demonstrated that both FBXO22 knockdown and FOXM1 knockdown dramatically reduced the level of FOXM1 at the Rad51 promoter (Fig. 5G and Fig. S6). Moreover, as a Fanconi gene (FANCR), Rad51 may also be indirectly regulated by FOXM1 in a transcriptional repressor DREAM-dependent manner [17, 18]. To clarify whether the DREAM complex is involved in the regulation of Rad51 by FBXO22 and FOXM1, we hindered the formation of the DREAM complex by using the Harmine inhibitor and silencing of LIN9 (the core component of DREAM), respectively. The results showed that neither Harmine inhibitor nor LIN9 silencing affect the downregulation of Rad51 caused by FBXO22 and FOXM1 knockdown, suggesting that the regulatory effects of FBXO22 and FOXM1 on Rad51 is independent of the DREAM complex (Fig. S7). Collectively, FBXO22 increases the level of FOXM1 at the Rad51 promoter, which in turn enhances the transcriptional activity of Rad51 and ultimately leads to lung cancer radioresistance.

### Deguelin is a potential FBXO22 inhibitor and radiotherapy sensitizer

**Fig. 6:**
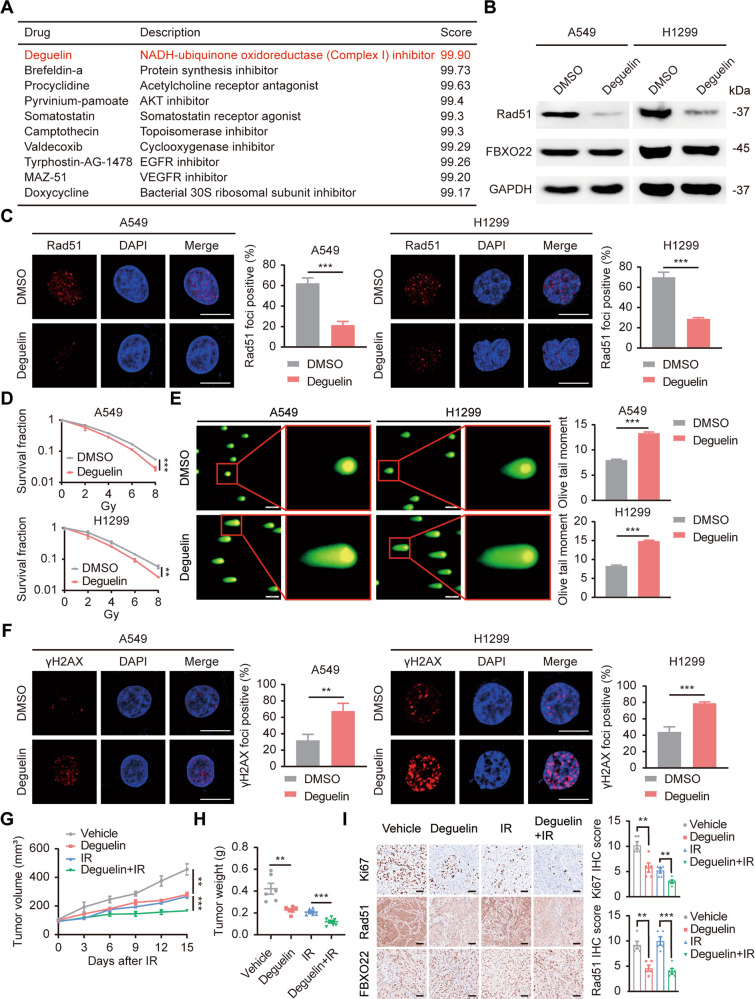
Deguelin is a potential FBXO22 inhibitor and radiotherapy sensitizer. **A** List of potential FBXO22 inhibitors obtained from the CMap database. **B** Rad51 expression was downregulated in deguelin-treated cells. **C** DMSO-treated and deguelin-treated cells were subjected to 6 Gy physical irradiation followed by Rad51 foci staining. Representative images and statistical histograms are presented. *** *P* < 0.001 (*n* = 3). Scale bar: 10 µm. **D** A colony formation assay was performed on DMSO-treated and deguelin-treated cells. The colonies were counted, and survival curves were plotted after 14 days. ** *P* < 0.01, *** *P* < 0.001 (*n* = 3). **E** Deguelin-treated cells had longer olive tails than DMSO-treated cells. The data are presented as the mean ± SEM. *** *P* < 0.001 (*n* = 100). Scale bar: 20 µm. **F** More γH2AX foci-positive cells were observed in deguelin-treated cells than in DMSO-treated cells. ** *P* < 0.01, *** *P* < 0.001 (*n* = 3). Scale bar: 10 µm. **G** The volumes of xenograft tumors were measured every 3 days, and growth curves were plotted. The data are presented as the mean ± SEM. ** *P* < 0.01, *** *P* < 0.001 (*n* = 6). **H** The tumor weights of each group were recorded and are shown as the mean ± SEM. ** *P* < 0.01, *** *P* < 0.001 (*n* = 6). **I** IHC staining for Ki67, Rad51, and FBXO22 was conducted on xenograft tumors in each group, and representative images and statistical histograms of IHC scores are presented. ** *P* < 0.01, *** *P* < 0.001 (*n* = 5).

Given the function of FBXO22 in lung cancer radioresistance, searching for drugs targeting FBXO22 has profound clinical significance. CMap (https://clue.io/) is an applicable database established using gene expression profiles of human cells treated with different interventions, including small molecule inhibitors [19]. A comparison of our RNA-seq data with the CMap database reference dataset yielded a list of candidate drugs with the highest correlation scores (Fig. 6A). These drugs produced gene expression profiles similar to those after FBXO22 silencing and were identified as potential inhibitors of FBXO22. Deguelin, ranked first, is a natural rotenoids derived from legumes and can exert antitumor and chemopreventive effects by targeting multiple oncogenic signaling pathways [20]. To define the effect of deguelin on the inhibition of FBXO22, we first measured its IC50 value in two lung cancer cell lines and selected 50 μM accordingly for subsequent experiments (Fig. S8A). qRT-PCR results showed that similar to the gene expression profile induced by FBXO22 deletion, deguelin also decreased the mRNA levels of HR molecules (Fig. S8B). Importantly, Rad51, the key downstream substrate of FBXO22, was also downregulated in deguelin-treated cells, suggesting that deguelin can inhibit the function of FBXO22 (Fig. 6B, C). Does deguelin exert a synergistic effect with radiotherapy? In vitro functional assays showed that deguelin-treated lung cancer cells had decreased colony formation ability, elongated comet tails, and an increased number of γH2AX foci, predicting elevated radiosensitivity of the cells (Fig. 6D–F). We further explored the in vivo effects of deguelin by establishing xenograft nude mouse models. The mice were orally administered deguelin or solvent every two days, and the tumor volumes were measured regularly. Our results suggested that deguelin slowed the growth of xenografts to some extent, while growth inhibition was most pronounced when deguelin was combined with radiotherapy (Fig. 6G, H and Fig. S9). This conclusion was further confirmed by Ki67 IHC staining (Fig. 6I). In addition, we monitored the in vivo safety of the combination treatment, and the results showed no significant difference in body weight gain, no remarkable pathological damage to important organs, and no significant abnormalities in peripheral blood biochemical parameters in each group (Fig. S10). Accordingly, deguelin is a potential inhibitor of FBXO22 and a safe and effective radiosensitizing agent for lung cancer radiotherapy.

### The radiosensitizing effect of deguelin is dependent on the functional inhibition of FBXO22

**Fig. 7:**
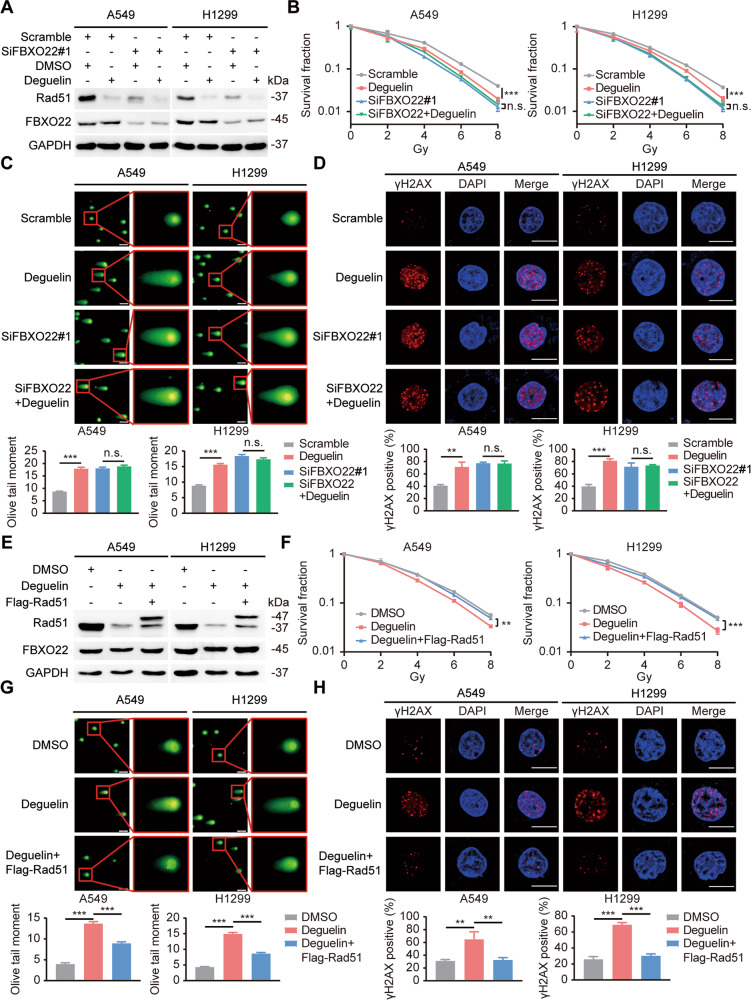
The radiosensitizing effect of deguelin is dependent on FBXO22 inhibition. **A** A549 and H1299 cells were transfected with scrambled siRNA or SiFBXO22 and treated with DMSO or deguelin for 24 h. Cells were collected and examined by Western blotting. **B** A colony formation assay was carried out, and the colonies were counted after 14 days. *** *P* < 0.001, n.s. *P* > 0.05 (*n* = 3). **C** Representative images of the neutral comet assay and statistical histograms of olive tail moments for each group are presented. The data are shown as the mean ± SEM. *** *P* < 0.001, n.s. *P* > 0.05 (*n* = 100). **D** Representative pictures of γH2AX foci staining for the indicated groups. ** *P* < 0.01, *** P < 0.001, n.s. *P* > 0.05 (*n* = 3). **E** A549 and H1299 cells were transfected with the indicated plasmids and treated with DMSO or deguelin for 24 h. Cells were collected and detected by Western blotting. **F-H** Colony formation assay, neutral comet assay, and γH2AX foci staining were used to evaluate the radiosensitivity of the indicated group of cells. ** *P* < 0.01, *** *P* < 0.001.

To further clarify the role of FBXO22 in deguelin function, wild-type and FBXO22-silenced lung cancer cells were treated with deguelin separately. Subsequent functional experiments showed that deguelin impaired the colony formation ability of wild-type cells after radiotherapy and exacerbated the radiation-induced DNA damage in the cells; however, it did not increase the radiosensitivity of FBXO22-deficient cells, indicating that the functional exertion of deguelin is dependent on FBXO22 (Fig. 7A–D). Importantly, we also evaluated the role of Rad51 in deguelin function. Consistent with the expected results, exogenous overexpression of Rad51 almost completely reversed the deguelin-mediated radiosensitization (Fig. 7E–H). Overall, our results demonstrate that deguelin can inhibit the function of FBXO22 and enhance lung cancer radiosensitivity in a Rad51-dependent manner.

## Discussion

In the present study, we found that FBXO22 was aberrantly highly expressed in lung cancer and could serve as a novel molecular biomarker of radioresistance. Mechanistically, FBXO22 promoted Rad51 gene transcription by increasing the level of FOXM1 at the Rad51 promoter, which contributes to insensitivity to lung cancer radiotherapy. For this reason, pharmacological targeting of FBXO22 is expected to be a promising strategy to improve the efficacy of radiotherapy. Deguelin was identified as a functional inhibitor of FBXO22 by utilizing the CMap database and was shown to increase lung cancer radiosensitivity in vitro and in vivo with a favorable safety profile, exhibiting substantial clinical translation prospects.

FBXO22 is the F-box receptor subunit of SCF E3 ubiquitin ligase. An increasing number of studies have shown that FBXO22 exerts oncogenic or suppressor effects in a variety of human tumors by targeting different substrate molecules [21]. The expression of FBXO22 has been reported to be upregulated in hepatocellular carcinoma, pancreatic carcinoma, and melanoma and downregulated in ER^+^HER2^-^ breast cancer and renal cell carcinoma [22, 23]. To reveal the clinical significance of FBXO22 in lung cancer, we examined the expression levels of FBXO22 in lung cancer cells and lung cancer tissues and analyzed the correlation between FBXO22 expression and the patients’ prognosis according to their survival information. The findings indicated that FBXO22 was remarkably overexpressed in lung cancer and that high expression of FBXO22 was correlated with short overall survival of patients, suggesting that FBXO22 is a biomarker of poor prognosis in lung cancer. Since FBXO22 influences the therapeutic response of solid tumors to DNA-damaging agents [13], we further explored the relationship between FBXO22 and lung cancer radiosensitivity. Our results showed that FBXO22-deficient lung cancer cells showed worse radiation-induced DNA damage and greater radiosensitivity than control cells. However, Sarmishtha De et al. reported that FBXO22 sensitized lung cancer cells to DNA-damaging agents by degrading PD-L1 [24]. There are several possible reasons for this discrepancy. First, Sarmishtha De et al. simply used PDL1 as a mediator to explain radiosensitivity, which may not have been sufficient. It should not be ignored that radiotherapy also produces immunosuppressive effects. Radiation induces upregulation of PDL1 expression and thus confers immunosuppression within the tumor, which can be reversed by FBXO22-mediated PDL1 degradation, resulting in a slowdown of growth in FBXO22-overexpressing cells. However, this observation is not truly due to radiosensitization. Unfortunately, we did not see the in vivo results obtained by the authors. In our study, targeted genetic or pharmacological inhibition of FBXO22 yielded significant radiotherapy-synergistic effects in mice. In addition, Sarmishtha De et al. used a single indicator of cell survival to measure radiosensitivity, which may have been inadequate since the effects of experimental factors on cell growth itself can greatly influence experimental findings. In our study, we used a colony formation assay to detect radiosensitivity, a gold standard method that excludes measurement bias arising from different cell proliferation rates [25]. Moreover, we examined the degree of radiation-induced DNA damage via a neutral comet assay and γH2AX foci staining, which indirectly reflected the radiosensitivity of tumor cells. The findings further confirmed our conclusion. Collectively, our findings indicate that overexpression of FBXO22 in lung cancer is a biomarker of poor prognosis and that FBXO22 silencing increases lung cancer radiosensitivity.

Radiotherapy causes DNA double-strand breaks, crossover, and ultimately apoptosis of tumor cells directly through radiation or indirectly through free radical production [26]. The inherent DNA damage repair mechanism in tumor cells is the main cause of radioresistance and diminished therapeutic effects [27]. HR is a central mechanism for accurately repairing DNA double-strand breaks, and excessive activation of HR leads to the insensitivity of tumor cells to radiotherapy [28]. Our transcriptome sequencing results showed that differentially expressed genes produced by FBXO22 silencing were significantly enriched in the HR pathway, indicating that overexpression of FBXO22 in lung cancer may induce radioresistance by activating the HR pathway. To determine the specific downstream substrates of FBXO22, we performed Western blotting on candidate HR molecules and found that the protein level of Rad51 was significantly decreased in FBXO22-deleted cells. Rad51 is the key catalyst of HR and is considered to act as the main guardian of genomic stability by facilitating accurate and efficient DNA repair [29]. Several studies have revealed the close relationship between Rad51 and radioresistance. Tumor patients with high Rad51 expression tend to experience poor radiotherapy response, whereas pharmacological inhibition of Rad51 dramatically increases radiosensitivity in prostate, breast, and lung cancers [30, 31]. Our rescue experiments showed that exogenous overexpression of Rad51 reversed the enhanced radiosensitivity caused by FBXO22 silencing, confirming the important role of Rad51 in FBXO22-induced radioresistance. Mechanistically, we found that the expression of FOXM1 was downregulated upon FBXO22 silencing and FOXM1 could promote Rad51 expression at both transcriptional and translational levels. The direct regulatory effect of FOXM1 on Rad51 was also confirmed by Luciferase assay and ChIP-PCR analysis. Moreover, the decreased expression of Rad51 and the enhanced radiosensitivity in FBXO22-deleted cells were attenuated by FOXM1 overexpression, and knockdown of FOXM1 also reversed the elevation in the expression of Rad51 and the insensitivity to radiation caused by FBXO22 overexpression. Therefore, we conclude that FBXO22 upregulates the expression of FOXM1 and FOXM1 directly binds to the Rad51 promoter to transactivate Rad51, which ultimately leads to lung cancer radioresistance. However, the specific way in which FBXO22 regulates FOXM1 remains unclear. It may be achieved through the ubiquitinated degradation of an intermediate molecule by FBXO22, or it may be related to the nonclassical function of FBXO22 independent of E3 ligase [32].

Considering the effect of FBXO22 in inducing lung cancer radioresistance, searching for FBXO22 inhibitors has important clinical applications. CMap is a gene expression profile database developed by the Broad Institute containing gene expression information from approximately 5000 small molecule compounds [19, 33]. We uploaded the 300 genes with the most significant expression changes due to FBXO22 silencing to the CMap database, which included 150 upregulated genes and 150 downregulated genes obtained from our RNA-seq data. A list of drugs that induce similar gene expression profiles and are thus potential inhibitors of FBXO22 was acquired. Deguelin was selected for subsequent experimental exploration because of its highest correlation score. As reported, deguelin is a natural rotenoids that is effective in reducing the incidence of tobacco-induced lung and mammary tumors in rats, showing chemopreventive potential [20, 34, 35]. In addition, deguelin exerts antitumor effects by targeting various oncogenic signaling pathways, whose functions include inhibition of tumor cell proliferation and metastasis, promotion of tumor cell apoptosis, arrest of the cell cycle, and induction of DNA damage [36–38]. Previously, Woo-Young Kim et al. reported that deguelin decreased the protein stability of HIF-1α by inhibiting HSP90, thereby increasing the radiosensitivity of radioresistant cell lines [39]. Our study further confirmed the radiosensitizing effect of deguelin in ex vivo lung cancer cells and proposed a novel mechanism to explain this phenomenon. In our results, deguelin induced an expression profile similar to that after FBXO22 silencing, leading to the downregulation of HR molecules, especially Rad51. Overexpression of Rad51 reversed deguelin-mediated radiosensitizing effects. Importantly, the knockdown of FBXO22 significantly reduced the responsiveness of lung cancer cells to deguelin, suggesting that deguelin functions in an FBXO22/Rad51-dependent manner. In summary, deguelin was identified as a small molecule inhibitor of FBXO22 that enhances lung cancer radiosensitivity in vitro and in vivo.

**Fig. 8:**
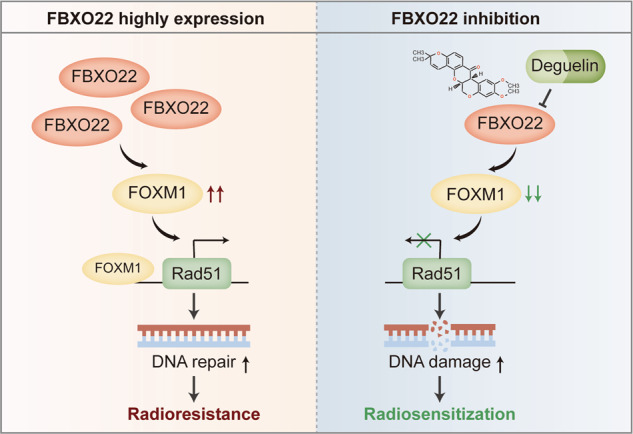
Schematic diagram showing the mechanism by which FBXO22 participates in lung cancer radioresistance. Overexpression of FBXO22 in lung cancer induces transcriptional activation of Rad51 by upregulating FOXM1, which promotes DNA damage repair and ultimately leads to lung cancer radioresistance. Deguelin was identified as an FBXO22 inhibitor that exerts radiosensitizing effects in vitro and in vivo, showing promising clinical translation prospects.

In conclusion, our study reveals a new biological function of FBXO22 in promoting lung cancer radioresistance. FBXO22 is aberrantly highly expressed in lung cancer and participates in radioresistance formation by activating the FOXM1/Rad51 axis. Deguelin is a potential inhibitor of FBXO22 that exerts radiosensitizing effects in vitro and in vivo with safe tolerance and is expected to be a promising radiosensitizer for clinical application (Fig. 8).

### Supplementary information

Supplementary Materials

Original Data

aj-checklist

## Data Availability

All data are available under reasonable request.
