# Supplementary material for: Targeting FBXO22 enhances radiosensitivity in non-small cell lung cancer by inhibiting the FOXM1/Rad51 axis
Source: Cell Death Dis. 2024 Jan 31;15(1):104. doi: 10.1038/s41419-024-06484-1 (PMC10830569; doi:10.1038/s41419-024-06484-1)
Supplement: Supplementary file 1 — Supplementary Materials [file 41419_2024_6484_MOESM1_ESM.pdf]

Genes	Sequences (5'--3')
FBXO22	F: TCGCATCTTACCACATACA
	R: TGATCCCATTGGAGTCAC
Rad51	F: GCTGATGAGTTTGGTGTAG
	R: TTTTGCAGATTCTGGTTT
TOPBP1	F: AGGTGGATTGGTGATAGAA
	R: CAAGGTAGGAGCGATGAA
XRCC3	F: CTGGCACTTGCTGAGAAC
	R: CAGTACGGGGACCTTCTT
BARD1	F: AGACACTAAGAGCAGGAATG
	R: AGAAATGGGACTGGAAAG
FOXM1	F: GCACGGCGGAAGATGAAG
	R: TGCTATGGCGGGCAAGCT
GAPDH	F: AGAAGGCTGGGGCTCATTTG
	R: AGGGGCCATCCACAGTCTTC

F, forward primer; R, reverse primer.

Table S1. Sequences of primers used for Real-time quantitative PCR.

Genes	Primer	**Sequences (5'--3')**
Rad51	Primer 1	F: GGAATGCGAGTAGGAGGCTC
		R: GGACTACATCTCCCGGCATG
	Primer 2	F: ACTCAGCCTTTAAAACCGGAATC
		R: GGAAGCTCGAGAAGATGGATAG
	Primer 3	F: CCGTGCAGGCCTTATATGATC
		R: ACAACACCACAAGCTAGCTG

F, forward primer; R, reverse primer.

Table S2. Sequences of primers used for ChIP-PCR.

Supplementary Figure and Figure Legends

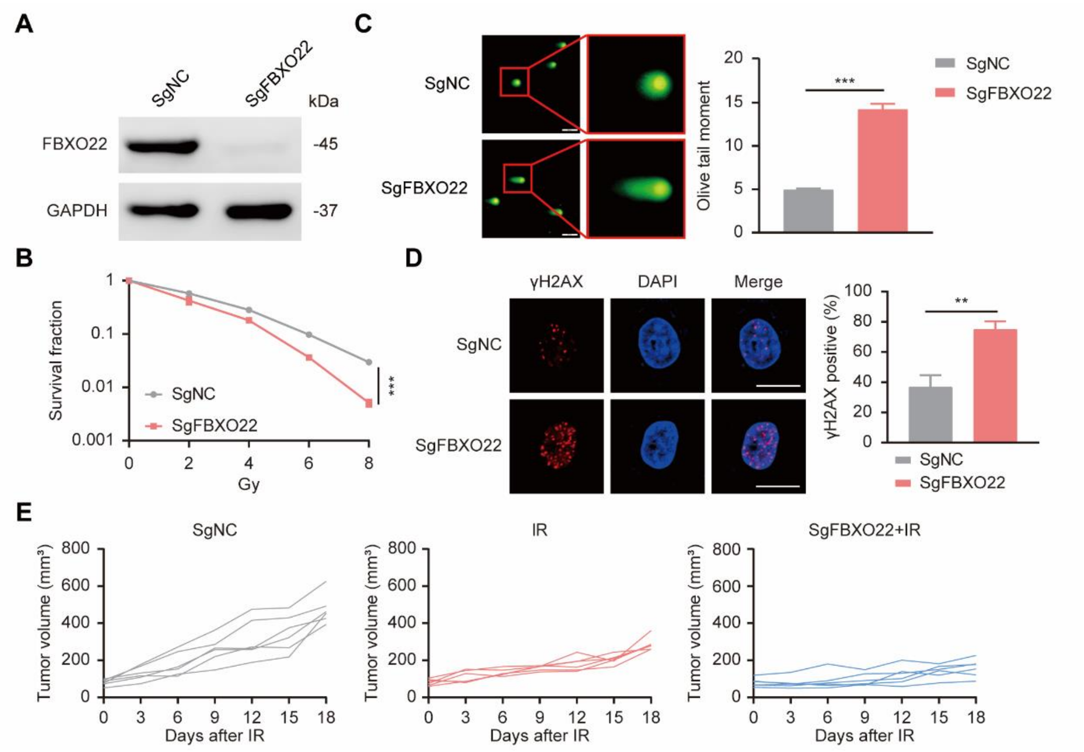

Fig. S1 FBXO22 knockout increases lung cancer radiosensitivity. A FBXO22 knockout cell line was successfully constructed by using CRISPR-Cas9 technology. **B** Cells with FBXO22 knockout had reduced survival fractions compared with the control group. *** $P < 0.001$ (n = 3). **C** FBXO22 knockout prolonged the olive tails of lung cancer cells after radiation. The data are presented as the mean ± SEM. *** $P < 0.001$ (n = 100). Scale bar: 20 µm. **D** More γH2AX foci were observed in sgFBXO22 cells compared with the control group. ** $P < 0.01$ (n = 3). Scale bar: 10 µm. **E** Tumor growth curves of individual mice were depicted (n = 6).

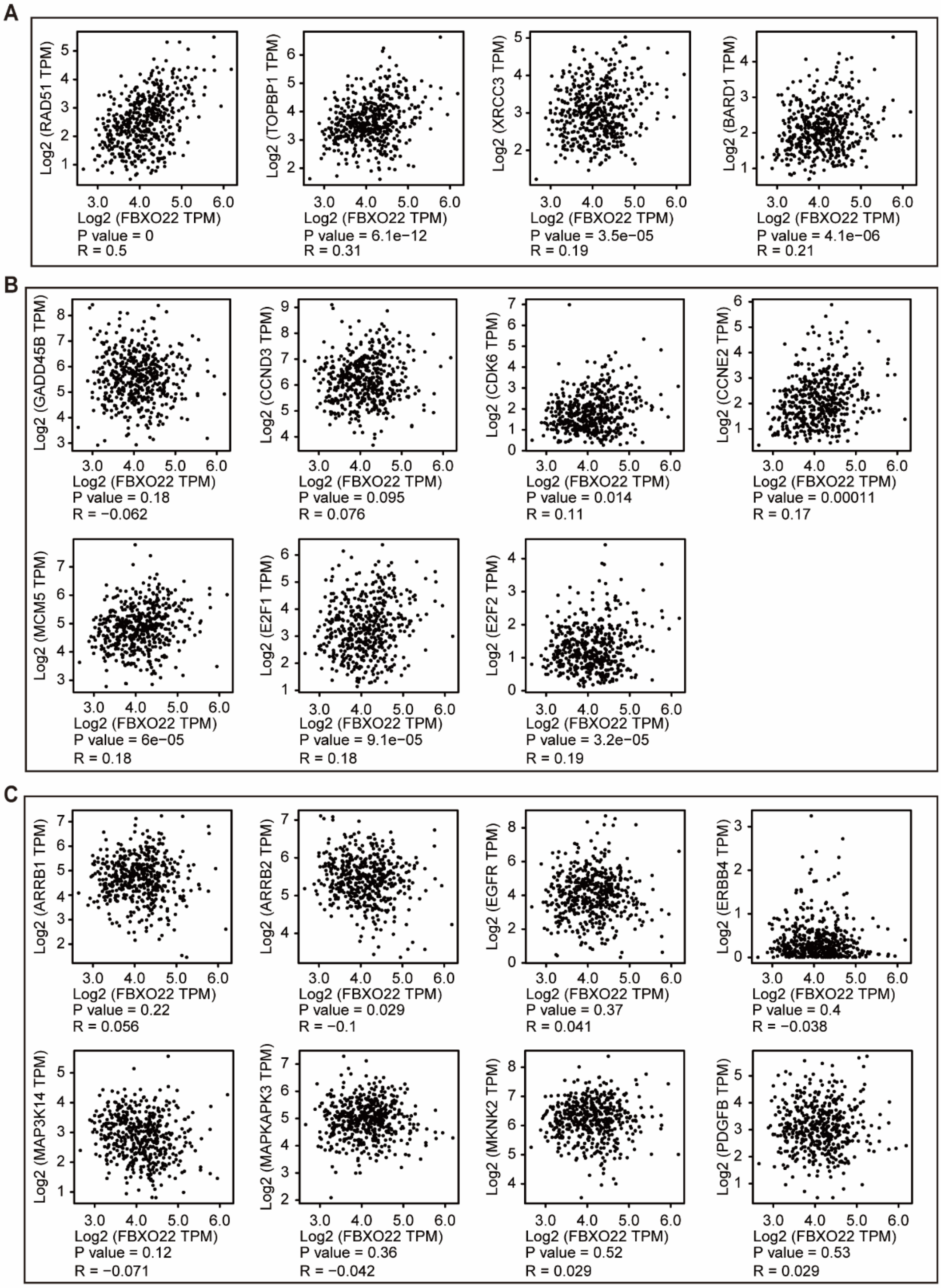

Fig. S2 Expression correlation between FBXO22 and molecules in homologous recombination, cell cycle, and MAPK signaling pathways in the GEPIA database. **A** HR molecules were all positively associated with FBXO22. **B** Molecules in the cell cycle pathway were either not correlated (*P* > 0.05) or weakly correlated (R < 0.2)

with FBXO22. **C** Molecules in the MAPK pathway were not correlated with FBOX22 (*P* > 0.05).

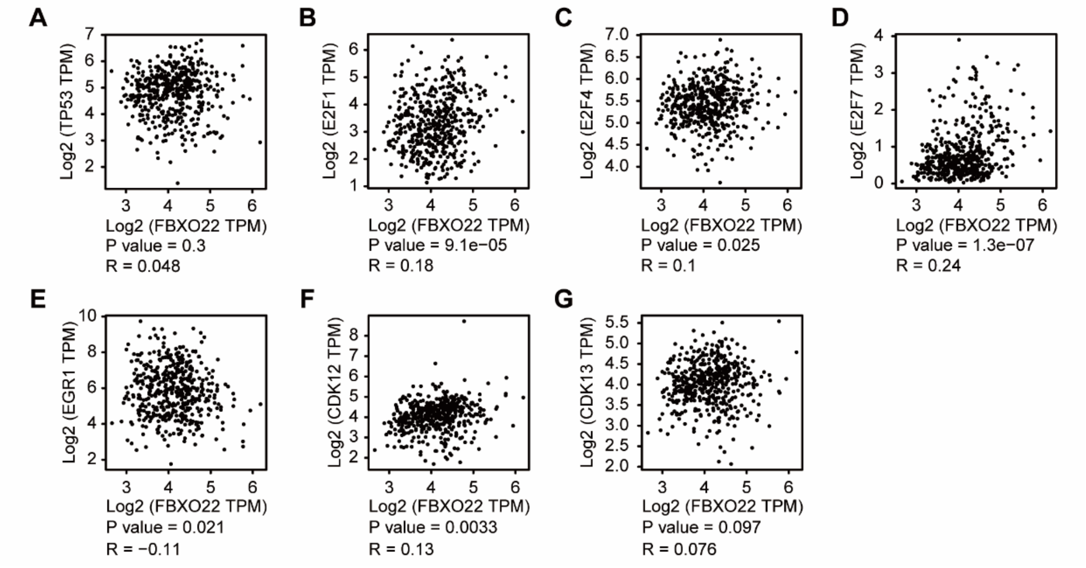

Fig. S3 Expression correlations between FBXO22 and Rad51 upstream transcription factors in the GEPIA database. A TP53. **B** E2F1. **C** E2F4. **D** E2F7. **E** EGR1. **F** CDK12. **G** CDK13.

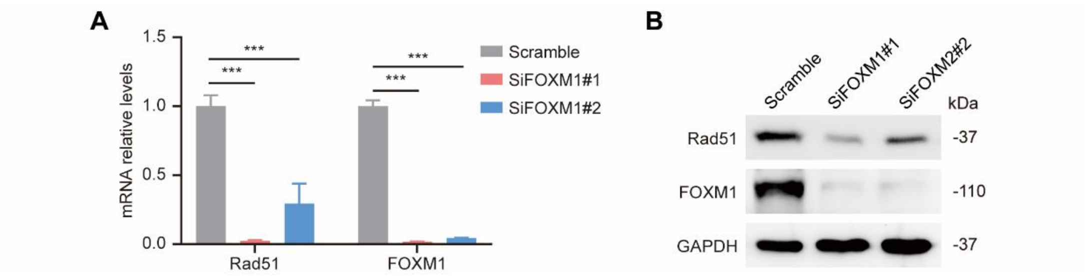

Fig. S4 Rad51 is positively regulated by FOXM1. A The mRNA level of Rad51 was examined by qRT-PCR in scrambled and FOXM1-deleted cells. ** *P* < 0.01, *** *P* < 0.001 (n = 4). **B** The protein level of Rad51 is reduced with FOXM1 silencing.

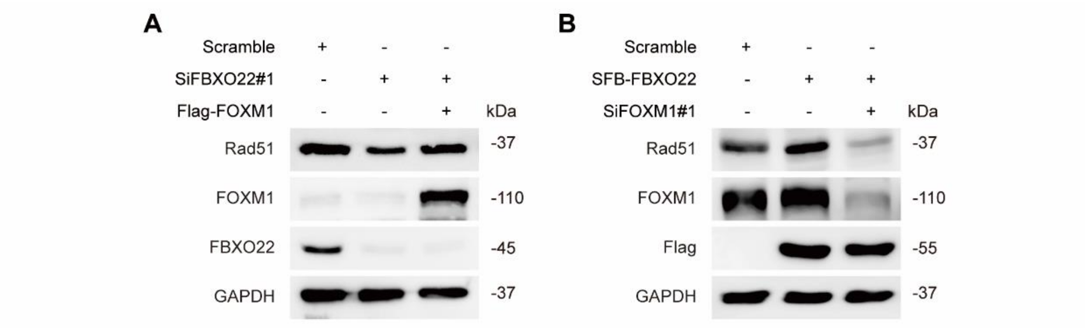

Fig. S5 FBXO22 regulates the expression of Rad51 in a FOXM1-dependent manner. A, B H1299 cells transfected with indicated siRNAs and plasmids were collected for Western blotting.

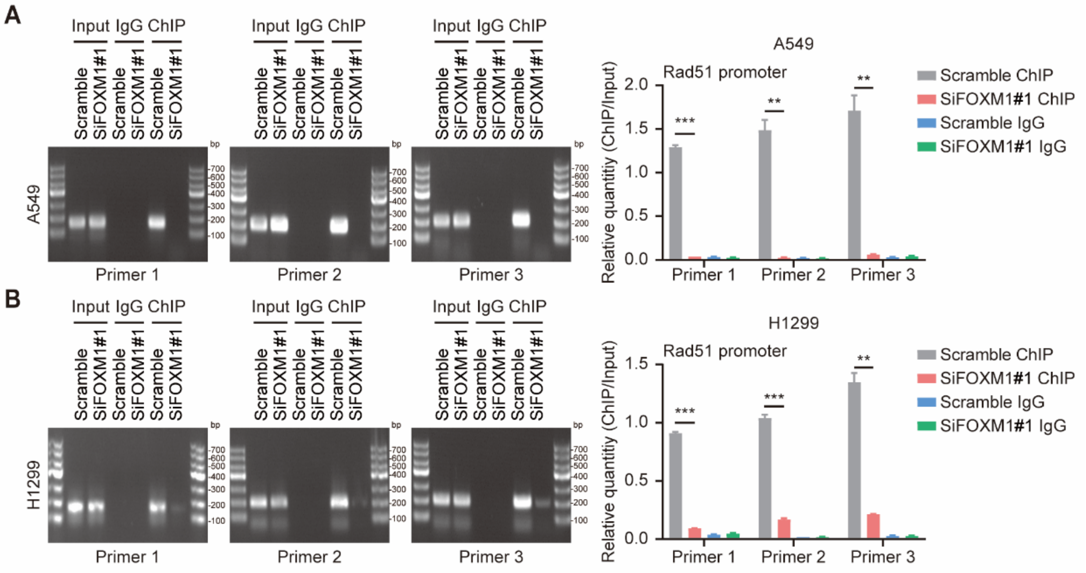

Fig. S6 Knockdown of FOXM1 significantly reduced the level of FOXM1 at the Rad51 promoter region. A A549. B H1299. ** $P < 0.01$, *** $P < 0.001$ (n = 3).

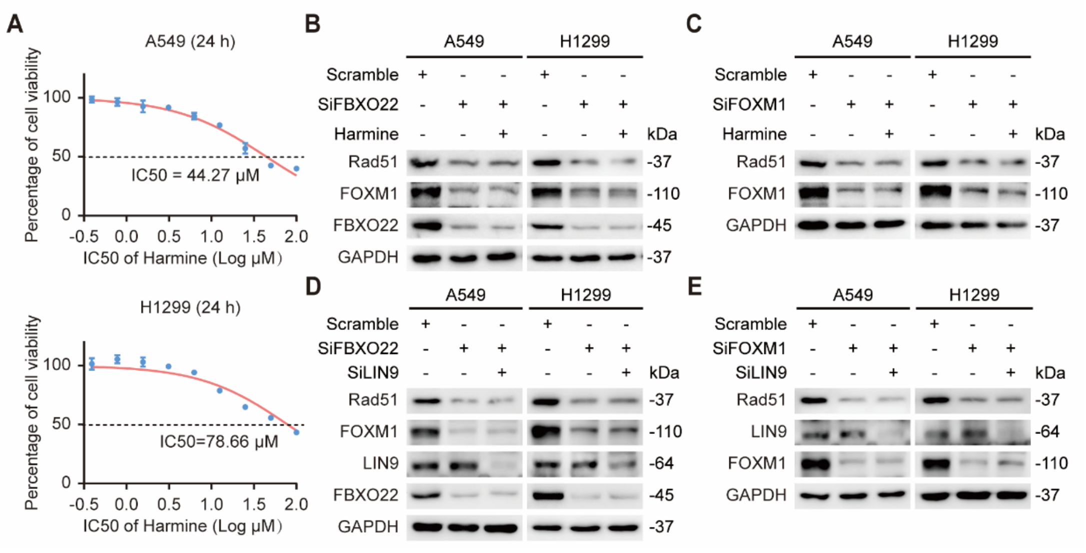

Fig. S7 FBXO22 and FOXM1 positively regulate the expression of Rad51 independently of the DREAM complex. A The IC50 (24 h) of Harmine in lung cancer cell lines was determined by CCK-8 assay. 40 μM and 75 μM were selected respectively in the A549 and H1299 cell lines. (n = 3). **B, C** A549 and H1299 Cells were transfected with the indicated siRNAs and treated with DMSO or Harmine for 24 h. Cells were collected and detected by Western blotting. **D, E** Cells transfected with the indicated siRNAs were harvested and analyzed by Western blotting.

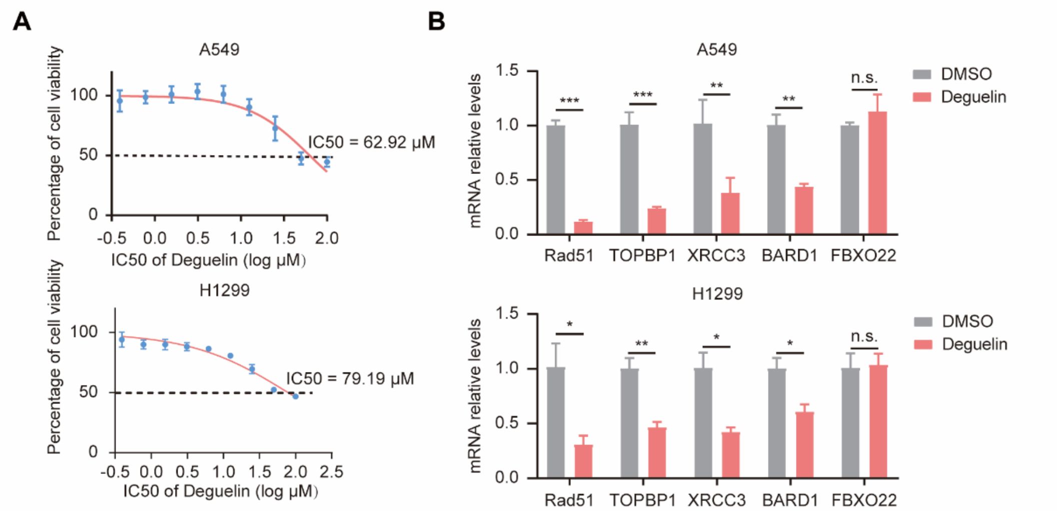

Fig. S8 Deguelin downregulated the expression of HR molecules. A The IC50 (24 h) of deguelin in A549 and H1299 cell lines was determined by CCK-8 assay, and 50 μM was selected accordingly for subsequent experiments in both cell lines (n = 3). **B** The mRNA levels of the indicated molecules were measured by qRT-PCR in DMSO-treated and deguelin-treated lung cancer cells. * $P< 0.05$, ** $P < 0.01$, *** $P < 0.001$, *n.s.* $P > 0.05$ (n = 4).

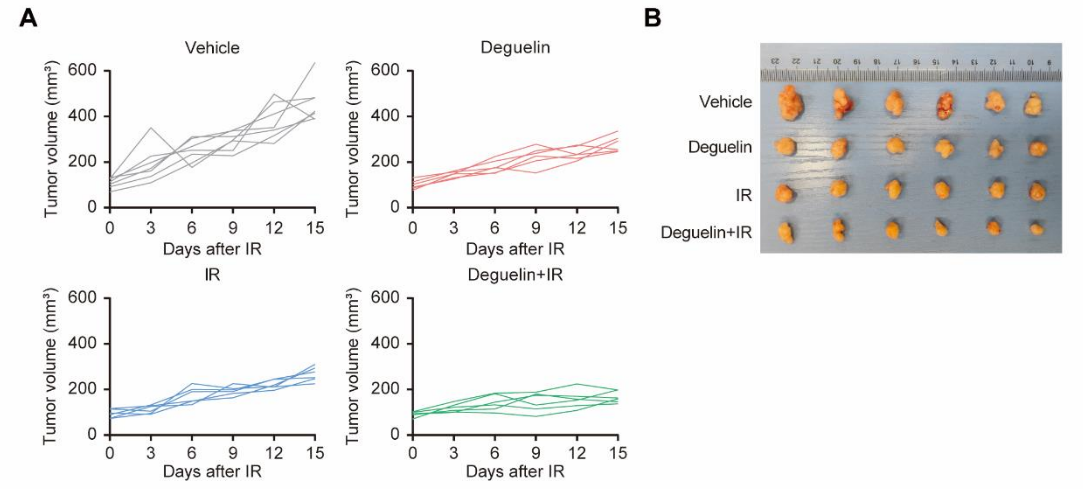

Fig. S9 Deguelin increases lung cancer radiosensitivity *in vivo*. A Tumor growth curves of individual mice were depicted (n = 6). **B** Xenograft tumors were removed and photographed when the experimental endpoint was reached (n = 6).

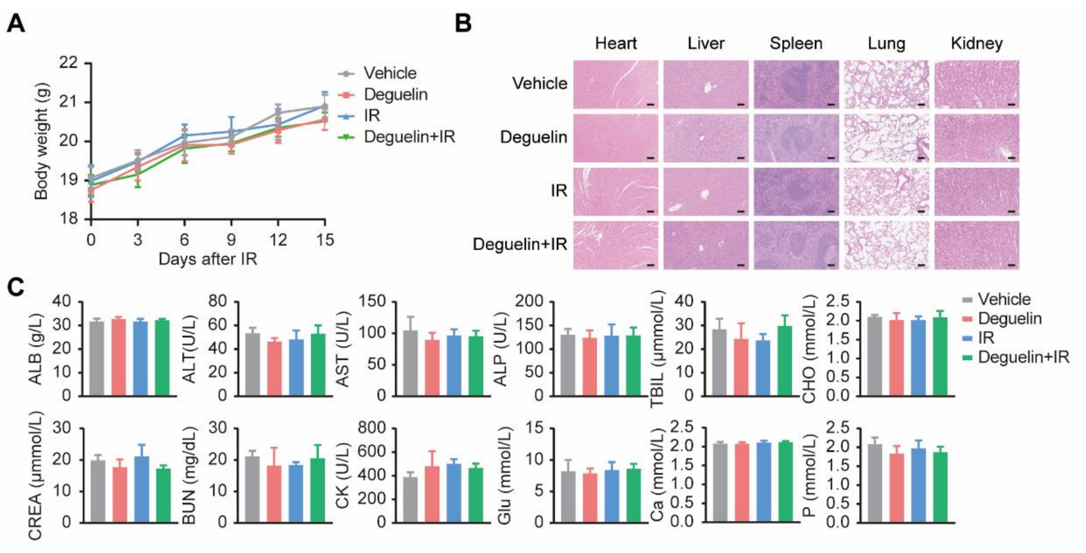

Fig. S10 The combination of deguelin and radiotherapy showed good safety *in vivo*. A Body weight growth curves for the different groups. The data are presented as the mean ± SEM (n = 6). **B** Representative HE staining images of important organs, including the heart, liver, spleen, lungs, and kidneys, of mice in each group. Scale bar: 100 μm. **C** The peripheral blood biochemical indices of mice were detected, and no significant abnormalities were found (n = 4). Abbreviations: ALB: Albumin; ALT: Glutamic aminotransferase; AST: Glutamic-oxaloacetic aminotransferase; ALP: Alkaline phosphatase; TBIL: Total bilirubin; CHO: Cholesterol; CERA: Creatinine; BUN: Urea nitrogen; CK: Creatine kinase; Glu: Glucose; Ca: Calcium; P: Inorganic phosphorus.
