# Supplementary figures and images for: Targeting FBXO22 enhances radiosensitivity in non-small cell lung cancer by inhibiting the FOXM1/Rad51 axis
Source: Cell Death Dis. 2024 Jan 31;15(1):104. doi: 10.1038/s41419-024-06484-1 (PMC10830569; doi:10.1038/s41419-024-06484-1)

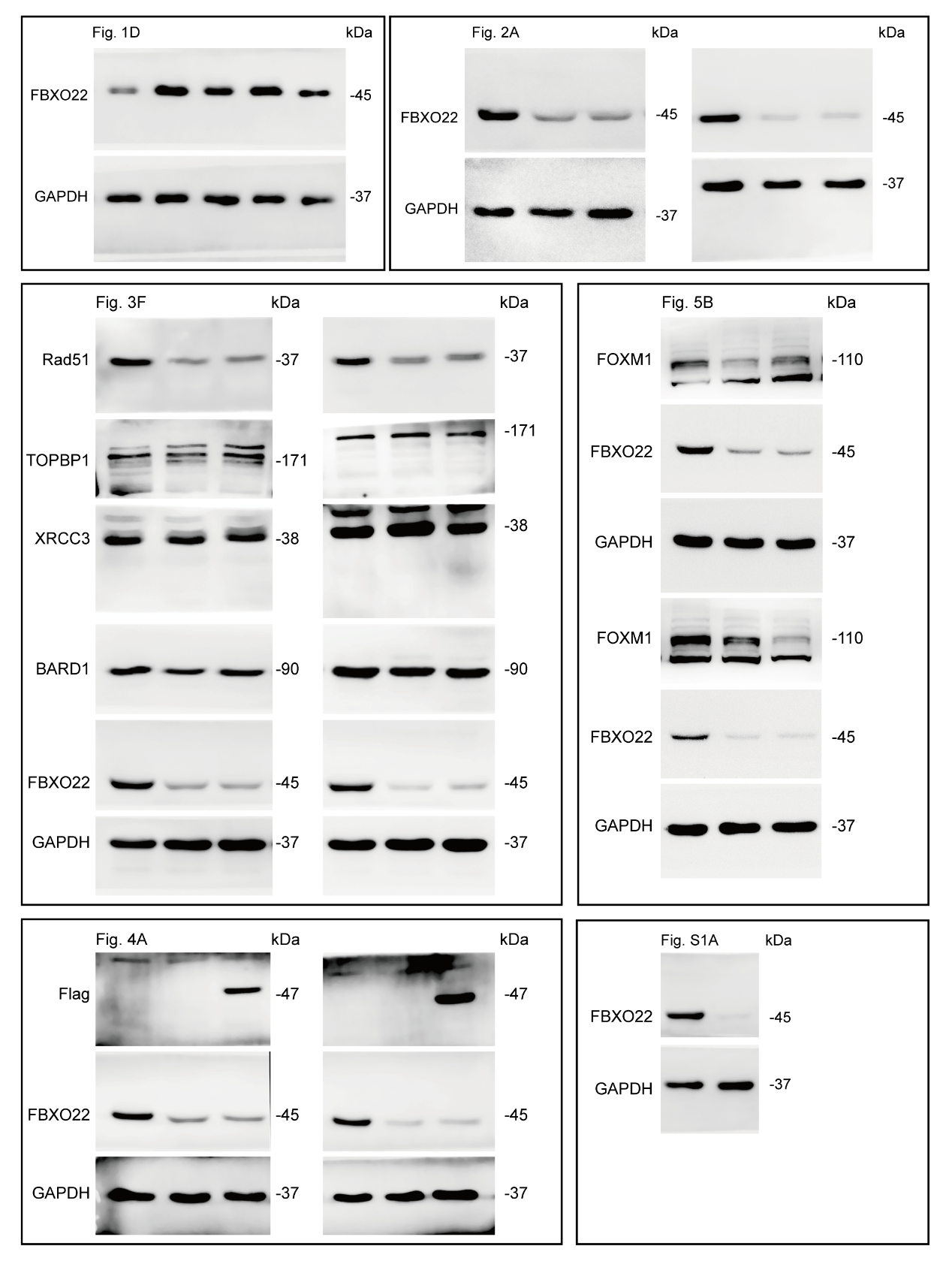

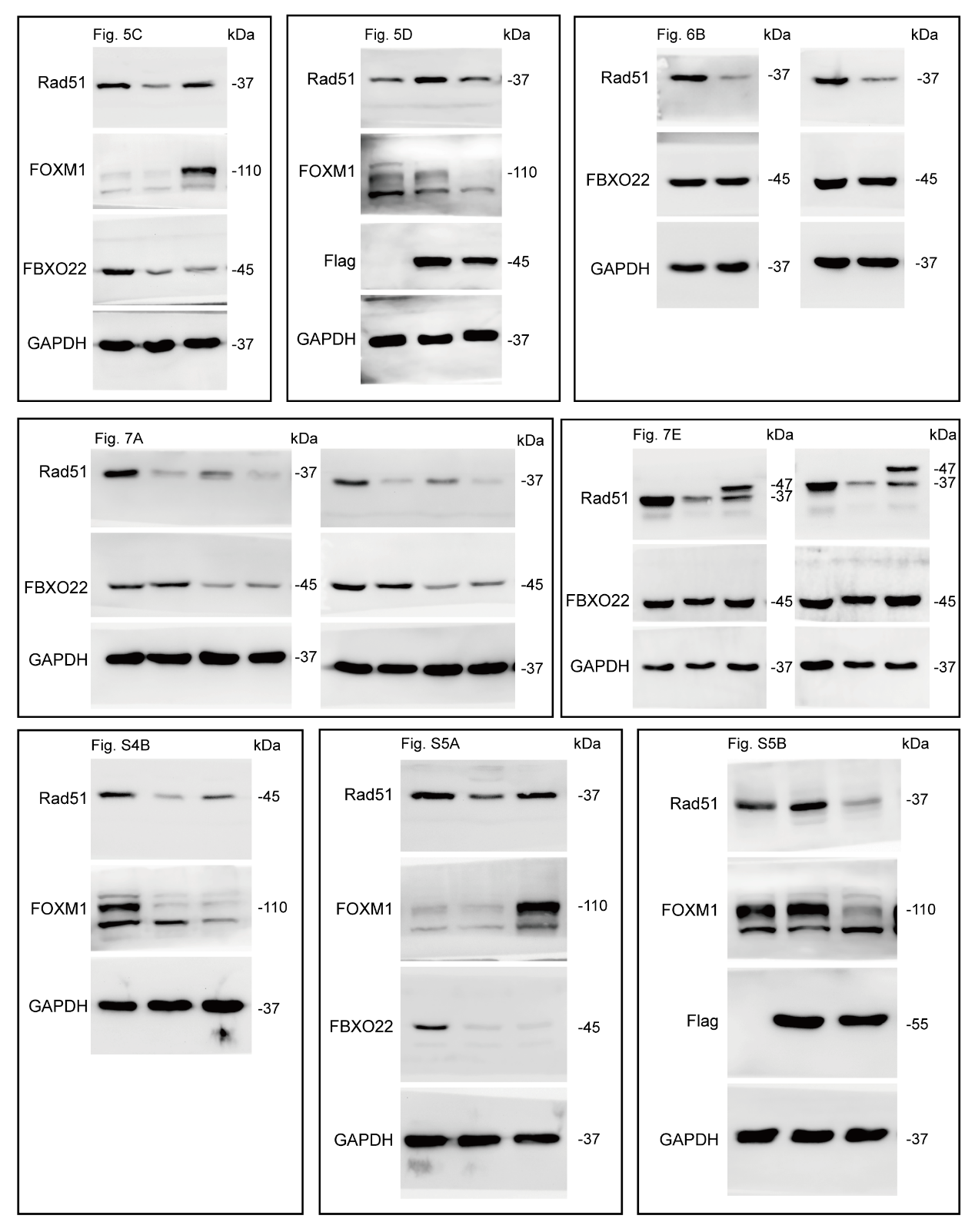

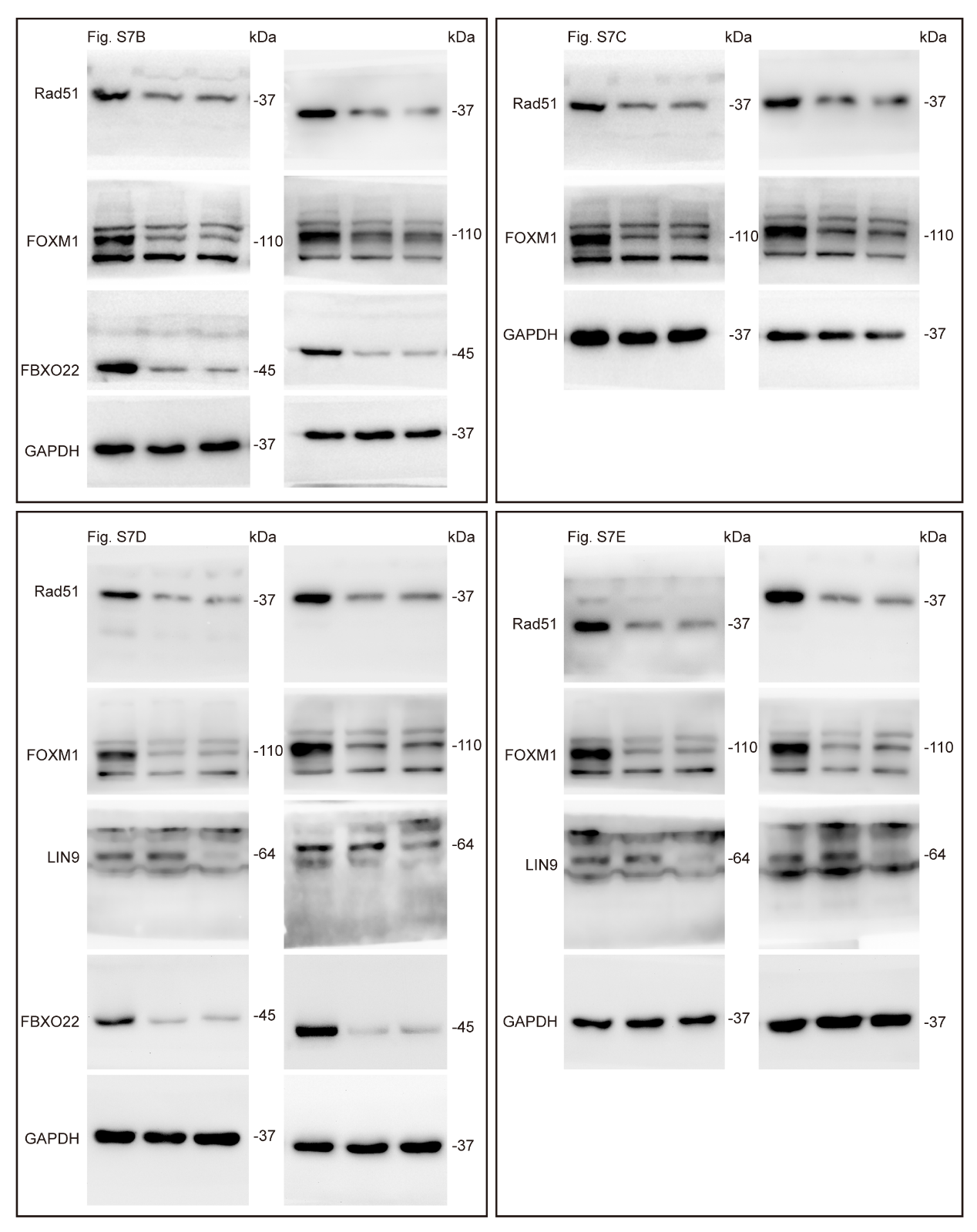

Supplement: Supplementary file 2 — Original Data [file 41419_2024_6484_MOESM2_ESM.docx]
